# Can implementation research be a game changer for measles elimination in Africa?

**DOI:** 10.11604/pamj.supp.2025.51.1.46913

**Published:** 2025-06-13

**Authors:** Abdu Abdullahi Adamu, Balcha Girma Masresha, Charles Shey Wiysonge

**Affiliations:** 1 Vaccine-Preventable Diseases Programme, World Health Organization, Regional Office for Africa, Djoue, BP 06, Brazzaville, Congo

**Keywords:** Measles, measles-containing vaccines, outbreaks, implementation research, Africa

## Abstract

Many countries in Africa still experience large and disruptive measles outbreaks despite the availability of a highly effective vaccine. In 2023, the estimated number of measles cases and deaths in the World Health Organization African Region was 4,801,946 cases and 75,942 deaths, respectively. Studies have shown that multiple implementation challenges affect equitable access and uptake of measles-containing vaccines, with specific barriers varying by settings. Implementation research, which emerged in response to know-do gaps in health systems, has several tools that can be used to gain a thorough understanding of the barriers to measles vaccination and how to address them using appropriate implementation strategies. In this commentary, we discuss why investment in implementation research for measles elimination is a programmatic imperative for countries in the WHO African Region.

## Commentary

Measles cases are rising in many countries worldwide despite the availability of a highly effective vaccine [1]. The World Health Organization (WHO) African Region disproportionately bears a high burden of measles, with an estimated 4,801,946 cases and 75,942 deaths in 2023 [1]. Vaccination coverage of at least 95% with two timely doses of measles-containing vaccines (MCV) is required to achieve herd immunity due to the infectiousness of the virus [1]. Despite efforts, many countries in the WHO African region lag in attaining the needed measles vaccination coverage benchmark [2]. According to the 2023 WHO - United Nations Children Fund (UNICEF) Estimate of National Immunization Coverage (WUENIC), coverage with first and second doses of measles-containing vaccines (MCV1 and MCV2) in the WHO African Region was 70% and 49%, respectively [2]. This is significantly below the threshold that is needed to achieve herd immunity and prevent recurrent outbreaks. Morbidity and mortality from measles are an unequivocal indication of social injustice, as the disease disproportionately affects poor, vulnerable, and underserved children.

Previous research has explored some of the complex, multifaceted, and multilevel contextual barriers that influence the implementation success of measles vaccination [3]. The dose-per-container presentation of the vaccine, the lyophilized nature of the vaccine, cold chain requirements, as well as the fact that it is injectable, present challenges for optimal administration by frontline health workers, as some may have limited training. Outer setting barriers are related to the socioeconomic status of the community and political and religious factors. Inner setting barriers are related to health facility operations, human resources for health capacity, and vaccine stockouts, among others. The contextual nature of the barriers to measles vaccination necessitates newer approaches to service delivery that foster the use of tailored strategies. This can advance meaningful progress toward improved and equitable measles vaccination coverage and, invariably, elimination. Implementation research offers useful tools that can aid a better understanding of how contextual barriers hinder routine uptake of health interventions and ways of systematically addressing them [4]. It involves recognizing, understanding, and addressing implementation bottlenecks, identifying optimal implementation options for a specific setting, and promoting the uptake of research findings into policy and practice [4]. Implementation research is not new, but unfortunately, it has not yet been widely used in immunization programmes to strengthen measles vaccination.

Over the years, tools and materials on implementation research have evolved, and expertise has expanded. What makes implementation research unique is its programme focus, which naturally necessitates closer collaboration and teaming between broad stakeholders (including frontline implementers and beneficiaries) compared to traditional research. There are several theory-informed frameworks (e.g., consolidated framework for implementation research (CFIR), theoretical domains framework (TDF), promoting action on research implementation in health services (PARIHS), etc.) for facilitating implementation research [5]. This type of research has been used across multiple programme areas such as malaria control, human immunodeficiency virus prevention, and tuberculosis care, among others, to improve the uptake of essential interventions [6]. Similarly, implementation research can improve measles vaccination in two main ways: promoting rapid use of new innovations and technology to alleviate access barriers to measles-containing vaccines in practice settings and optimizing measles-containing vaccine uptake at the delivery level through adaptive learning.

There is a general consensus on the need for enhanced vaccine delivery practices to achieve measles elimination goals, and this has motivated investments in various innovations [7]. Joseph Durlak, an eminent implementation scientist, once said, “When it comes to implementation, what is worth doing is worth doing well” [8]. This statement is particularly relevant in the context of emerging innovation for measles vaccination. It is a reminder that implementation evidence is crucial to the success of these innovations in impacting population health. One such innovation is the microneedle array patch for measles vaccination, which can overcome difficult programmatic bottlenecks that limit vaccination efforts in underserved areas [9]. Adigweme and colleagues, in their Phase I/II randomized trial in The Gambia, provided evidence that measles-containing vaccine microneedle array patches are safe for use and induce sufficient immunogenicity to protect against measles in children [9]. To minimize a “know-do” gap for this crucial innovation, it is pertinent that Phase III trials incorporate innovative design features that will enable researchers to answer real-world implementation-related questions. Early consideration for practical implementation is paramount for the trial evidence to pave its way faster for translation into regular practice.

Another innovation is the smaller number of doses per vial for measles-containing vaccines, which has huge potential to increase coverage while reducing wastage, and with only marginal cost increases [10]. There is ample evidence in countries using 10-dose vials that measles vaccination is not provided in every immunization session as a result of fear of vaccine wastage, especially in vaccination session settings with a small number of clients [3,10]. This is an example of missed opportunities created by the health system [3]. Although the use of smaller dose vials is accompanied by higher cost and logistics implications, their potential programmatic benefits make them worth considering, especially in targeted settings where client loads are less predictable, like rural hard-to-reach areas [10]. Implementation studies on using smaller-dose vials of measles-containing vaccines in specific contexts can provide useful “bottom-up” evidence to guide policies for their scale-up and spread. In a study in Zambia, a 5% increase in MCV1 coverage and a 3.5% increase in MCV2 coverage were detected in districts using 5-dose vials, and the vaccine wastage rate was 47% lower in facilities using 5-dose vials [10]. Implementation research can also be used to optimize performance at the service delivery level to improve the utilization of measles-containing vaccines. This can be achieved through context assessment and testing of practical solutions. The potential advantage of transforming problem-solving practices in delivery systems for routine immunization from “business as usual” to “adaptive learning” holds enormous promise not just for measles vaccination but also for other routine vaccines.

However, there are some enablers that immunization programmes need to unlock to benefit from the full potential of implementation research. Firstly, there is a need to build and sustain partnerships with researchers and academic institutions that have the capacity to conduct implementation research. This is important to ensure scientific rigor for the pragmatic research methods needed in implementation research. This can also ensure that the immunization program stays on track with its intention of conducting implementation research, frames the problem and objectives accordingly, and does not deviate into general health services research. Secondly, collaborative leadership is important to maintain full and purposeful participation among the different stakeholders with varying power dynamics involved in the implementation research endeavor. Thirdly, the use of implementation research in immunization programmes should be incorporated into existing policies and levers, which include but are not limited to streamlined ethical and regulatory processes, funding allocations, and incentivization.

In conclusion, implementation research can accelerate the use of new innovations for delivering measles-containing vaccines in routine settings, support the scale-up and spread of underutilized innovations, and optimize service delivery to improve measles vaccination. There is an urgent need to galvanize more attention for implementation research in immunization, especially for measles elimination, and mobilize resources to support its institutionalization in programme settings. Given the constantly increasing complexity of contexts and how it worsens inequity in measles vaccination coverage, immunization programmes can no longer afford to delay taking advantage of implementation research. It is time to rightfully position implementation research as a critical vehicle for operationalizing and advancing the strategic framework for measles elimination in the WHO African Region.
